# Developing and testing a framework for coding general practitioners’ free-text diagnoses in electronic medical records - a reliability study for generating training data in natural language processing

**DOI:** 10.1186/s12875-024-02514-1

**Published:** 2024-07-16

**Authors:** Audrey Wallnöfer, Jakob M. Burgstaller, Katja Weiss, Thomas Rosemann, Oliver Senn, Stefan Markun

**Affiliations:** Institute of primary care, University and University Hospital Zurich, Pestalozzistr. 24, Zürich, 8091 Switzerland

**Keywords:** General practitioners, Electronic medical records, Diagnostic coding, Reliability, Training data

## Abstract

**Background:**

Diagnoses entered by general practitioners into electronic medical records have great potential for research and practice, but unfortunately, diagnoses are often in uncoded format, making them of little use. Natural language processing (NLP) could assist in coding free-text diagnoses, but NLP models require local training data to unlock their potential. The aim of this study was to develop a framework of research-relevant diagnostic codes, to test the framework using free-text diagnoses from a Swiss primary care database and to generate training data for NLP modelling.

**Methods:**

The framework of diagnostic codes was developed based on input from local stakeholders and consideration of epidemiological data. After pre-testing, the framework contained 105 diagnostic codes, which were then applied by two raters who independently coded randomly drawn lines of free text (LoFT) from diagnosis lists extracted from the electronic medical records of 3000 patients of 27 general practitioners. Coding frequency and mean occurrence rates (n and %) and inter-rater reliability (IRR) of coding were calculated using Cohen’s kappa (Κ).

**Results:**

The sample consisted of 26,980 LoFT and in 56.3% no code could be assigned because it was not a specific diagnosis. The most common diagnostic codes were, ‘dorsopathies’ (3.9%, a code covering all types of back problems, including non-specific lower back pain, scoliosis, and others) and ‘other diseases of the circulatory system’ (3.1%). Raters were in almost perfect agreement (Κ ≥ 0.81) for 69 of the 105 diagnostic codes, and 28 codes showed a substantial agreement (K between 0.61 and 0.80). Both high coding frequency and almost perfect agreement were found in 37 codes, including codes that are particularly difficult to identify from components of the electronic medical record, such as musculoskeletal conditions, cancer or tobacco use.

**Conclusion:**

The coding framework was characterised by a subset of very frequent and highly reliable diagnostic codes, which will be the most valuable targets for training NLP models for automated disease classification based on free-text diagnoses from Swiss general practice.

**Supplementary Information:**

The online version contains supplementary material available at 10.1186/s12875-024-02514-1.

## Background

Routine data from primary care services can importantly contribute to health services research and other monitoring activities. In Switzerland, primary care is predominantly delivered by general practitioners (GPs), and 70% of the population visits a GP at least once a year [1]. Importantly for research and monitoring, the majority of healthcare contacts take place in this setting of care [2, 3]. Diagnostic data compiled by GPs is therefore a potential ressource for research and monitoring [4–8]. However, for statistical synthesis, diagnostic data requires coding [9]. Unfortunately, due to time pressure and the complexity of coding frameworks, diagnostic coding is very difficult to implement properly by GPs and there is no financial incentive for diagnostic coding in outpatients in Switzerland [10–12]. Thus, coded diagnoses are scarce for reasearch and monitoring in Swiss primary care.

The increasing use of electronic medical records by GPs makes data increasingly accessible for research, with even greater potential if coded diagnoses were readily available [13–16]. As a result, there is a need to advance the diagnostic coding of diagnoses obtained from GPs. Various methods can be used to achieve this, including purpose-built classification systems for primary care, such as the ICPC-2 code (*International Classification of Primary Care*,* 2nd edition*) [10, 12, 17–19]. However, the ICPC-2 code classifies reasons for encounters on a consultation level, which does not necessarily correspond to all diagnoses present, potentially leading to corresponding underestimation in epidemiological studies. The Systematized Nomenclature of Medicine Clinical Terms (SNOMED CT) is an other potential cadidate among coding systems which is designed to support documentation in electronic medical records and used in many major healthcare systems worldwide [20]. The SNOMED CT, is highly comprehensive containing over 2.5 million unique terms that describe not only diagnoses but a large range of clinical content comprised in a complex hierarchy that may be a limitation to coding reliablity [21].

The most widely used system for diagnostic coding is the ICD-10 (*10th revision of the International Statistical Classification of Diseases and Related Health Problems*) [22]. The ICD-10 is a classification system introduced by the World Health Organisation and serves as a global standard for identifying and reporting diseases and health conditions. It allows methodical documentation of disorders and diseases, injuries and other related health conditions and is interoperable with SNOMED CT [23]. The ICD-10, however, differentiates almost 70,000 diagnoses in a highly granulated fashion, making the system very precise but also very difficult to apply for inexperienced raters and it is therefore hardly suitable for coding by GPs [10, 17, 24].

Artificial intelligence applications from the domain of natural language processing (NLP) have substantially improved in recent years, are increasingly available and have great potential to support diagnostic coding in medicine [25–27]. However, to maximize their effectiveness, NLP models require training ideally on local and sufficiently sized and accurately labelled data, which may be scarce depending on healthcare setting [28]. In Swiss general practice, this challenge is particularly difficult for reasons explained above. In addition, even if GPs were to code their diagnoses, the accuracy of coding would still be highly uncertain, given the paucity of training and lack of incentives GPs have in this domain. In order to face this challenge of lacking training data from Swiss general practice, we aimed to develop a framework of relevant diagnostic codes, apply it to a dataset and measure the frequency of codes as well as the reliability of coding, which will be relevant for further using the data for NLP training.

## Methods

### Study design, setting and ethics statement

This was a study of frequency and inter-rater reliability (IRR) in diagnostic coding using a purposely-developed coding framework in a large primary care database. To select the diagnostic codes, we harvested opinions from local stakeholders as well as epidemiological data to emphasize both the local relevance of codes and expected prevalence of diagnoses in this setting. The large primary care database involved was the FIRE database (FIRE stands for “Family Medicine Research using Electronic Medical Records”), which contains anonymized patient data from Swiss GPs’ electronic medical records [29]. Specifically, the database holds almost nine million consultation records from over 500 Swiss GPs including medication prescription data, clinical parameters, results from laboratory tests, as well as coded reasons for encounters. In addition, the database holds administrative data and sufficiently truncated demographic information from patients to enable anonymization. Patient identifiers are anonymized via a GP-sided hashing procedure enabling identification of patients by a numeric code without breaking anonymity. The availability of the unstructured free text format ‘diagnoses and problems lists’ is a recently introduced feature of the FIRE database that made the present study possible. The local Ethics Committee of the Canton of Zurich waived approval for research with the FIRE database because patient data is fully anonymized and therefore outside the scope of the Swiss Human Research Act (BASEC-Nr. Req2017–00797). The study was conducted in accordance with the Declaration of Helsinki and good clinical practice guidelines.

### Diagnostic codes

We pre-specified that the number of different diagnostic codes should be limited to approximately 100 in order to prevent over-dispersion. To take relevance for local stakeholders into account, 4 stakeholders (JB, LJ, OS, AP) independently compiled a list of diagnostic codes they deemed relevant to their research. To achieve our goal we tasked these experts with up-coding the original ICD-10 classification (ICD-10 origin) to the highest level of the code that still was meaningful to them. Unused codes from each ICD-10 chapter were grouped together into a code range containing the remaining diseases for the respective chapter. To consider the expected prevalence of diagnoses in general practice, we used four previously published lists of the 100 most frequent ICD-10 diagnoses in general practice from Nordrhein-Westfalen (NRW-lists), each list covering consecutive three-month periods ranging between the second quarter of 2021 and the first quarter of 2022 [30–33]. Diagnostic codes were directly selected for the subsequent coding process if at least three out of four stakeholders independently proposed to include them. Additionally, we included codes proposed by two stakeholders if additionally appearing on each NRW-list. Codes that were proposed by only one or two stakeholders and also appeared on each of the four NRW-lists were subjected to a second committee of stakeholders (SM, AP, AW, KW) who rated the importance of each code to their research on a scale from 1 (lowest importance) to 3 (highest importance). Codes achieving at least 5 points were added to the selection diagnostic codes used in the subsequent coding process ultimately consisting of 115 different codes.

### Data selection, coding process and analysis

For this study, we used data from 27 GPs nested in 10 different general practices. Specifically, from each practice, we randomly drew 300 patients with at least one consultation in the year 2019. From these patients, we used the patient ID and the contents of the “diagnosis and problems lists” which are text fields to insert according information in free-text format from patients’ last consultation in 2019, as imputed by the GPs. This data was transferred into a spreadsheet where each line of free-text (LoFT) from the electronic medical record was assigned to an individual cell using only line breaks (or formatting information to the same effect depending on electronic medical record software) for parsing. A pre-testing subset containing 10% of the LoFT was drawn to test the intended coding process and refine the coding framework where necessary. Pre-testing revealed redundancies and very low occurrence (that is zero occurrences) of specific codes, which were subsequently unified or removed from the selection and thus, the final coding framework consisted of 105 different codes which served as rulebook for subsequent coding (see Additional File 1).

The coding process involved two trained physicians (AW and DB) who were tasked to independently assign the diagnostic codes to each LoFT. Raters were tasked to code every LoFT, which reflected an unambiguous diagnosis (that is the unambiguous name of a diagnosis corresponding to a diagnostic code from the framework in the absence of qualifying statements or diagnostic considerations indicating a relevant diagnostic uncertainty). In the event of ambiguity or information insufficient to code a diagnosis (such as LoFT describing mere symptoms, laboratory test results or low certainty differential diagnostic considerations) the code for “no diagnosis” was assigned, so that every LoFT in the dataset was coded. Such a “no diagnosis” code was necessary because free-text fields are notorious for non-specific data overflow in electronic medical records and a NLP model will heavily depend on accurate identification of such data [34].

In all of the LoFT, we determined for each diagnostic code: (1) frequency by rater, (2) average occurrence rate (as percentage) using the total count of LoFT as denominator and the respective code as numerator, (3) inter-rater agreement (IRA) using the total count of LoFT as denominator and the count of LoFT with concordant coding (absence or presence of the respective code) of the respective code as numerator and (4) inter-rater reliability (IRR) using Cohen’s kappa as measure [35]. We used counts and proportions (n and %) for descriptive statistics. We interpreted Κ ≥ 0.81 as almost perfect agreement K between 0.61 and 0.80 as substantial agreement. For data analysis, we used the software R (Version 4.2.0) [36].

## Results

### Sample and frequency analyses

The random sample of 3000 patients was 55.2% female, the mean age was 52.2 (SD 21.4) years. From these patients, we obtained 26,980 LoFT (of which 2,800 were used for pre-testing). To the 26,980 LoFT, raters 1 and 2 assigned 31,672 and 31,864 codes respectively (the number of codes exceeded the number of LoFT because of cases where multiple codes were assigned to a single LoFT). Taken together, raters most frequently assigned diagnostic codes: “no diagnosis” (56.3%), “dorsopathies” (3.9%), “other diseases of the circulatory system” (3.1%,) and “other diseases of the musculoskeletal system and connective tissue” (2.8%). A frequency of at least 200 (0.7% of LoFT) by at least one rater was encountered in 30 codes (see Table 1) and a frequency of at least 100 (0.4%) was encountered in 51 codes. Eleven codes were assigned with a frequency below 30 (0.1%) by either rater (see Additional File 2 for the complete frequency analysis).

**Table 1:** The thirty most frequently assigned codes or code ranges

ICD-10-Origin	Code	Rater 1	Rater 2	Avg. of LoFT%	Kappa
none	no diagnosis	15,300	15,091	56.3%	0.856
M40-M54	dorsopathies	1056	1066	3.9%	0.932
I00-I99	other diseases of the circulatory system	824	865	3.1%	0.848
M00-M99	other diseases of the musculoskeletal system and connective tissue	769	758	2.8%	0.743
I10	primary hypertension	713	704	2.6%	0.972
S00-T98	injury, poisoning and certain other consequences of external causes	654	690	2.5%	0.853
D00-D48	other neoplasms	581	588	2.2%	0.852
E78	disorders of lipoprotein metabolism and other lipidaemias	545	539	2.0%	0.985
E00-E90	other endocrine, nutritional and metabolic diseases	489	501	1.8%	0.876
M60-M79	soft tissue disorders	415	463	1.6%	0.734
K00-K93	other diseases of the digestive system	414	449	1.6%	0.786
L00-L99	other diseases of the skin and subcutaneous tissue	401	458	1.6%	0.833
H00-H59	diseases of the eye and adnexa	344	350	1.3%	0.900
C00-C99	malignant neoplasms	333	359	1.3%	0.839
F17	mental and behavioural disorders due to use of tobacco	312	315	1.2%	0.969
I20-I25	ischaemic heart diseases	297	305	1.1%	0.925
K57	diverticular disease of intestine	284	281	1.0%	0.973
N00-N99	other diseases of the genitourinary system	252	302	1.0%	0.780
K21	gastro-oesophageal reflux disease	262	260	1.0%	0.957
E65-E68	obesity and other hyperalimentation	260	260	1.0%	0.961
G00-G99	other diseases of the nervous system	234	263	0.9%	0.778
F32-F33	depressive episode and recurrent depressive disorder	238	249	0.9%	0.96
E00-E07	disorders of thyroid gland	223	238	0.9%	0.883
J00-J99	other diseases of the respiratory system	212	247	0.9%	0.751
A00-B99	intestinal infectious diseases	236	218	0.8%	0.785
K40-K46	hernia	221	221	0.8%	0.950
H60-H95	other diseases of the ear and mastoid process	226	207	0.8%	0.862
E11	type 2 diabetes mellitus	217	211	0.8%	0.906
I83	varicose veins of lower extremities	195	217	0.8%	0.882
D50-D90	other diseases of the blood and blood-forming organs and certain disorders involving the immune mechanism	183	207	0.7%	0.782

### Agreement and reliability

With respect to measures of coding agreement, we found IRA of > 0.98 in all assigned codes except “no diagnosis” (IRA = 0.93). With respect to IRR, we found Kappa values ≥ 0.810 in 69 of all the 105 diagnostic codes and 28 codes showed Kappa between 0.610 and < 0.810. Simultaneously a frequency of 100 by at least one rater and a Kappa value ≥ 0.81 was found in 37 codes (see Table 2). Among these frequently assigned diagnostic codes, we found the highest IRR in “disorders of lipoprotein metabolism and other lipidaemias” (Kappa = 0.985), “diverticular disease of intestine” (Kappa = 0.973) and “primary hypertension” (Kappa = 0.972).

**Table 2:** Codes that were both frequently and reliably assigned

ICD-10 -Origin	Code	Rater 1	Rater 2	Avg. of LoFT%	Kappa
E78	disorders of lipoprotein metabolism and other lipidaemias	545	539	2.0%	0.985
K57	diverticular disease of intestine	284	281	1.0%	0.973
I10	primary hypertension	713	704	2.6%	0.972
F17	mental and behavioural disorders due to use of tobacco	312	315	1.2%	0.969
I11-I14	hypertension with end organ damage	163	159	0.6%	0.962
I48	atrial fibrillation and flutter	147	140	0.5%	0.961
E65-E68	obesity and other hyperalimentation	260	260	1.0%	0.961
E55	vitamin D deficiency	112	113	0.4%	0.960
F32-F33	depressive episode and recurrent depressive disorder	238	249	0.9%	0.960
K21	gastro-oesophageal reflux disease	262	260	1.0%	0.957
N18	chronic kidney disease	110	108	0.4%	0.954
M17	arthritis of the knee	183	176	0.7%	0.952
N40	hyperplasia of prostate	106	115	0.4%	0.950
K40-K46	intestinal hernia	221	221	0.8%	0.950
G47	sleep disorders	150	147	0.6%	0.949
J45	asthma	174	177	0.7%	0.945
M40-M54	dorsopathies	1056	1066	3.9%	0.932
I20-I25	ischaemic heart diseases	297	305	1.1%	0.925
K64	haemorrhoids and perianal venous thrombosis	122	119	0.4%	0.921
K29	gastritis and duodenitis	143	150	0.5%	0.914
E11	type 2 diabetes mellitus	217	211	0.8%	0.906
H00-H59	diseases of the eye and adnexa	344	350	1.3%	0.900
N80-N98	noninflammatory disorders of female genital tract	133	128	0.5%	0.896
E00-E07	disorders of thyroid gland	223	238	0.9%	0.883
I83	varicose veins of lower extremities	195	217	0.8%	0.882
E00-E90	other endocrine, nutritional and metabolic diseases	489	501	1.8%	0.876
I60-I69	cerebrovascular diseases	135	130	0.5%	0.874
H60-H95	other diseases of the ear and mastoid process	226	207	0.8%	0.862
no diagnosis	no diagnosis	15,300	15,091	56.3%	0.856
S00-T98	injury, poisoning and certain other consequences of external causes	654	690	2.5%	0.853
D00-D48	other neoplasms	581	588	2.2%	0.852
I00-I99	other diseases of the circulatory system	824	865	3.1%	0.848
F40-F48	neurotic, stress-related and somatoform disorders	162	197	0.7%	0.839
C00-C99	malignant neoplasms	333	359	1.3%	0.839
L00-L99	other diseases of the skin and subcutaneous tissue	401	458	1.6%	0.833

## Discussion

Obtaining coded diagnoses from Swiss GP is difficult but necessary for training NLP models. In this study, we developed a set of 105 diagnostic codes, applied them to a moderately sized dataset of only about 26,000 LoFT and measured frequencies as well as reliability of codes. Over a third of the codes achieved both a frequency above 100 and an almost perfect IRR and are thus suitable for training NLP models using this dataset. The most promising codes in this regard are those that are not easily identified by methods using other data from the electronic medical record (such as laboratory tests or disease-specific medications) and LoFT are the only data source, such as musculoskeletal conditions, cancer or tobacco use.

We developed diagnostic codes with the a priori intention of generating training data for NLP models. To do this, we attempted to limit the granularity of the diagnostic codes to around 100 items in order to avoid over-dispersion, where rarely occurring codes would have insufficient frequency to train NLP models on moderately sized datasets. Within the set of coded LoFT, 51 codes were assigned at least 100 times by both raters and are therefore potential candidates for exploring the feasibility of NLP. Interestingly, however, more than half of the LoFT were coded as ‘no diagnosis’, suggesting that GPs use this space for additional information that does not amount to a specific diagnosis. This is consistent with findings from other studies that have analysed the content of LoFT, showing that non-specific or insufficient information is common in medical documentation [34, 37–39] but substantially reduced the yield of LoFT for obtaining coded diagnostic data in our study. Specifically, ambiguous acronyms or abbreviations [40–42], unstructured information [42–44], as well as physicians’ and institutional stylistic preferences contribute to non-diagnostic information in free-text diagnoses [45]. Raters in our study were notably challenged by non-diagnostic information in LoFT, which manifested itself in an IRA of only 93%, whereas all other codes had IRA ≥ 98%. We strongly expect that these difficulties will be transferred to the NLP modelling process and methods will be needed to deal not only with false positive identifications but also with ambiguity within the LoFT itself. Third party review and arbitration can be used to further process the training data, but such human arbitration is arguably not a perfect gold standard and may inevitably introduce bias in addition to that introduced when the LoFT was created. This chain of fundamental validity issues highlights important future limitations of NLP-identified diagnoses and foreseeably questions the feasibility of fully automated coding in cases where very high accuracy is required.

Unsurprisingly, the most frequently assigned diagnostic codes were those for the most common chronic or recurrent conditions, particularly those of the musculoskeletal and cardiovascular systems [46]. Several of these diagnoses were already identifiable in the FIRE database based on algorithms applied to routine data such as prescribed medications (e.g., antidiabetic drugs to identify diabetes) or results of clinical or laboratory tests (e.g., body mass index for obesity) [47]. However, there are several important and prevalent diagnoses for which sufficiently specific identification criteria based on routine data are lacking, including musculoskeletal conditions, cancer, tobacco use, depression, sleep disorders and many others, which are important targets of research in general practice. These diagnoses represent the area where we expect NLP to add the most value for research using the FIRE database.

It can be assumed that the data from the FIRE database are representative of the general practice setting in Switzerland [48]. In this study, although limited to 10 practices and 27 GPs, the representativeness of the sample is supported by the fact that the patients were almost identical in demographic characteristics compared to a recent epidemiologic study that also sampled consecutive patients in Swiss general practice [46]. In terms of code frequency, the rankings of the codes seemed plausible, as they correspond to the rankings of disease prevalence estimates in the Swiss population. Specifically, dorsopathies, followed by essential hypertension and hyperlipidemia, are the most frequently appearing chronic diseases in this setting according to external studies [49–54]. Moreover, frequencies in our study are also very similar to a study measuring reasons for encounters in general practice where diseases of the musculoskeletal and cardio-circulatory systems were by far the most prevalent, thus adding to the plausibility of our results [55–57].

With regard to IRR, we observed almost perfect agreement (Kappa ≥ 0.810) in two thirds of the codes and substantial agreement in another quarter. Taken together, more than 90% of codes had at least substantial agreement when rated by raters having completed medical school without further training. These findings are comparatively favorable when similar studies with inexperienced raters are considered [24, 58, 59] and equal to studies with experienced raters [60]. Depending on the research question and the target diseases to be coded, Kappa values ≥ 0.500 are generally deemed sufficient [35, 58, 61] and thus, the codes we developed appeared to perform sufficiently. Previous studies have shown that code frequency is associated with IRR [62, 63]. This finding was replicated in our study, where all of the 20 most frequent codes reached either almost perfect or substantial IRR, while the 20 least frequent codes had a Kappa ≤ 0.600.

### Strengths and limitations

This research project describes the design and reliability testing of a custom coding framework to be used for training NLP models. The project can serve as a template for similar research, which will become increasingly important given the growing role of AI in medicine and the associated need for local training data tailored to local factors such as languages and use cases. The use of LoFT from general practice-based medical diagnosis lists is a very prominent use case in this regard, and our study provides estimates of code frequencies based on a moderately sized dataset, which can be achieved with a small investment in manual coding labor. The methods used are highly feasible and provide transparent metrics that help in further interpretation of NLP modelling results, especially when considering the IRR of coding by human raters labelling the training data.

The moderate size and locality of the dataset may be a major limitation. We tried to include LoFT data from a representative sample of Swiss GPs, but this sample still only included 27 of them, and these were nested in 10 different medical practices. The local jargon of these GPs may limit the applicability of NLP models based on these training data. The jargon used by Swiss general practitioners may be particularly heterogeneous given the fact that Switzerland has four different languages in close proximity to each other and is also subject to a high level of international immigration of health professionals from completely different linguistic regions. Furthermore, while IRR serves to determine the degree of agreement between raters it does not necessarily measure accuracy. Therefore even after disagreement was solved, our data may still contain mislabeled LoFT conveying biases from the independent raters which will impact training of NLP models. Therefore, NLP models will need to undergo rigorous testing and external validation, and the quality of the training data itself may need to be improved.

## Conclusion

We developed and tested a framework of research-relevant diagnostic codes in a primary care research database to train NLP models based on free text data. We have identified a subset of very frequent and highly reliable diagnostic codes, and the next step in the research agenda is to train NLP models with the obtained data and evaluate their performance in automated disease classification.

### Electronic supplementary material

Below is the link to the electronic supplementary material.

Supplementary Material 1

Supplementary Material 2

## Data Availability

Data for this study was provided by the FIRE project (FIRE stands for “Family Medicine Research using Electronic Medical Records”), which gathers anonymized patient data from Swiss general practitioners’ electronic medical records.Data is not publicly available but can be obtained upon reasonable request (for contact information see: https://www.fireproject.ch/en).

## Abbreviations

FIRE: Family Medicine ICPC Research using Electronic Medical Records
LoFT: Lines of free-text
GP: General Practitioner
ICD-10: International Statistical Classification of Diseases and Related Health Problems, Tenth Revision
IRA: Interrater agreement
IRR: Interrater reliability
