# Supplementary material for: Developing and testing a framework for coding general practitioners’ free-text diagnoses in electronic medical records - a reliability study for generating training data in natural language processing
Source: BMC Prim Care. 2024 Jul 16;25:257. doi: 10.1186/s12875-024-02514-1 (PMC11251376; doi:10.1186/s12875-024-02514-1)
Supplement: Supplementary file 1 — Supplementary Material 1 [file 12875_2024_2514_MOESM1_ESM.docx]

Additional file 1: Coding Framework

| **Category** | **ICD-Origin** | **Framework code** |
| --- | --- | --- |
| Infectious | | |
|  | [A00-B99](https://www.icd-code.de/icd/code/A00-B99.html) | other intestinal infectious diseases |
|  | B00 | herpes simplex infections |
|  | [B02](https://www.icd-code.de/icd/code/B02.-.html) | herpes zoster |
|  | [B15-B19](https://www.icd-code.de/icd/code/B15-B19.html) | viral hepatitis |
|  | [B20-B24](https://www.icd-code.de/icd/code/B20-B24.html) | human immunodeficiency virus disease |
| Neoplastic | | |
|  | C00-C99 | malignant neoplasms |
|  | D00-D48 | other neoplasms |
| Blood formation | | |
|  | [D50](https://www.icd-code.de/icd/code/D50.-.html) | iron deficiency anaemia |
|  | [D50-D90](https://www.icd-code.de/icd/code/D50-D90.html) | other diseases of the blood and blood-forming organs and certain disorders involving the immune mechanism |
| Endocrine | | |
|  | [E00-E07](https://www.icd-code.de/icd/code/E00-E07.html) | disorders of thyroid gland |
|  | E05 | hyperthyroidism |
|  | [E10](https://www.icd-code.de/icd/code/E10.-.html) | type 1 diabetes mellitus |
|  | [E11](https://www.icd-code.de/icd/code/E11.-.html) | type 2 diabetes mellitus |
|  | E14 | unspecified diabetes mellitus |
|  | E55 | vitamin D deficiency |
|  | [E65-E68](https://www.icd-code.de/icd/code/E65-E68.html) | obesity and other hyperalimentation |
|  | E78 | disorders of lipoprotein metabolism and other lipidaemias |
|  | [E00-E90](https://www.icd-code.de/icd/code/E00-E90.html) | other endocrine, nutritional and metabolic diseases |
| Psychiatric | | |
|  | F00-F03 | unspecified dementia |
|  | [F10-F19](https://www.icd-code.de/icd/code/F11.-.html) | mental and behavioural disorders due to use of alcohol |
|  | [F10](https://www.icd-code.de/icd/code/F10.-.html) | mental and behavioural disorders due to psychoactive substance use |
|  | [F17](https://www.icd-code.de/icd/code/F17.-.html) | mental and behavioural disorders due to use of tobacco |
|  | [F20-F29](https://www.icd-code.de/icd/code/F20-F29.html) | schizophrenia, schizotypal and delusional disorders |
|  | [F30-F39](https://www.icd-code.de/icd/code/F30-F39.html) | affective disorders |
|  | F32 | depressive episode and recurrent depressive disorder |
|  | F40-F48 | neurotic, stress-related and somatoform disorders |
|  | F50 | eating disorders |
|  | [F00-F99](https://www.icd-code.de/icd/code/F00-F99.html) | other mental and behavioural disorders |
| Neurological | | |
|  | [G20-G26](https://www.icd-code.de/icd/code/G20-G26.html) | extrapyramidal and movement disorders |
|  | G20 | parkinson's disease |
|  | [G35](https://www.icd-code.de/icd/code/G35.-.html) | multiple sclerosis |
|  | [G40](https://www.icd-code.de/icd/code/G40.-.html) | epilepsy |
|  | [G43](https://www.icd-code.de/icd/code/G43.-.html) | migraine |
|  | [G45](https://www.icd-code.de/icd/code/G45.-.html) | transient cerebral ischaemic attacks and related syndromes |
|  | [G47](https://www.icd-code.de/icd/code/G47.-.html) | sleep disorders |
|  | [G60-G64](https://www.icd-code.de/icd/code/G60-G64.html) | polyneuropathies and other disorders of the peripheral nervous system |
|  | [G44](https://www.icd-code.de/icd/code/G44.-.html) | other headache syndromes |
|  | [G00-G99](https://www.icd-code.de/icd/code/G00-G99.html) | other diseases of the nervous system |
| Eye | | |
|  | [H00-H59](https://www.icd-code.de/icd/code/H00-H59.html) | diseases of the eye and adnexa |
| Ear | | |
|  | [H65](https://www.icd-code.de/icd/code/H65.-.html) | nonsuppurative otitis media |
|  | [H66](https://www.icd-code.de/icd/code/H66.-.html) | suppurative and unspecified otitis media |
|  | [H81](https://www.icd-code.de/icd/code/H81.-.html) | disorders of vestibular function |
|  | [H60-H95](https://www.icd-code.de/icd/code/H60-H95.html) | other diseases of the ear and mastoid process |
| Circulation | | |
|  | [I10](https://www.icd-code.de/icd/code/I10.-.html) | primary hypertension |
|  | [I11-I14](https://www.icd-code.de/icd/code/I11.-.html) | hypertension with end organ damage |
|  | [I15](https://www.icd-code.de/icd/code/I15.-.html) | secondary hypertension |
|  | [I20-I25](https://www.icd-code.de/icd/code/I20-I25.html) | ischaemic heart diseases |
|  | I34 | nonrheumatic mitral valve disorders |
|  | [I48](https://www.icd-code.de/icd/code/I48.-.html) | atrial fibrillation and flutter |
|  | [I50](https://www.icd-code.de/icd/code/I50.-.html) | heart failure |
|  | [I60-I69](https://www.icd-code.de/icd/code/I60-I69.html) | cerebrovascular diseases |
|  | I70 | atherosclerosis |
|  | [I83](https://www.icd-code.de/icd/code/I83.-.html) | varicose veins of lower extremities |
|  | [I87](https://www.icd-code.de/icd/code/I87.-.html) | other disorders of veins |
|  | [I00-I99](https://www.icd-code.de/icd/code/I00-I99.html) | other diseases of the circulatory system |
| Respiration | | |
|  | [J00-J06](https://www.icd-code.de/icd/code/J00-J06.html) | acute upper respiratory infections |
|  | J02 | acute pharyngitis |
|  | [J09-J18](https://www.icd-code.de/icd/code/J09-J18.html) | influenza and pneumonia |
|  | [J20-J22](https://www.icd-code.de/icd/code/J20-J22.html) | other acute lower respiratory infections |
|  | J30 | vasomotor and allergic rhinitis |
|  | [J45](https://www.icd-code.de/icd/code/J45.-.html) | asthma |
|  | [J44](https://www.icd-code.de/icd/code/J44.-.html) | other chronic obstructive pulmonary disease |
|  | [J00-J99](https://www.icd-code.de/icd/code/J00-J99.html) | other diseases of the respiratory system |
| Gastro-intestinal | | |
|  | [K21](https://www.icd-code.de/icd/code/K21.-.html) | gastro-oesophageal reflux disease |
|  | K25-K28 | peptic ulcer |
|  | [K29](https://www.icd-code.de/icd/code/K29.-.html) | gastritis and duodenitis |
|  | [K30](https://www.icd-code.de/icd/code/K30.html) | functional dyspepsia |
|  | [K40-K46](https://www.icd-code.de/icd/code/K40-K46.html) | hernia |
|  | [K50-K52](https://www.icd-code.de/icd/code/K50-K52.html) | noninfective enteritis and colitis |
|  | [K57](https://www.icd-code.de/icd/code/K57.-.html) | diverticular disease of intestine |
|  | [K58](https://www.icd-code.de/icd/code/K58.-.html) | irritable bowel syndrome |
|  | [K64](https://www.icd-code.de/icd/code/K64.-.html) | haemorrhoids and perianal venous thrombosis |
|  | [K70-K77](https://www.icd-code.de/icd/code/K70-K77.html) | diseases of liver |
|  | [K80-K87](https://www.icd-code.de/icd/code/K80-K87.html) | disorders of gallbladder, biliary tract and pancreas |
|  | [K00-K93](https://www.icd-code.de/icd/code/K00-K93.html) | other diseases of the digestive system |
| Skin | | |
|  | L40 | psoriasis |
|  | [L00-L99](https://www.icd-code.de/icd/code/L00-L99.html) | other diseases of the skin and subcutaneous tissue |
| Musculoskeletal | | |
|  | M10 | gout |
|  | M15 | polyarthritis |
|  | M16 | arthritis of the hip |
|  | M17 | arthritis of the knee |
|  | [M18-M19](https://www.icd-code.de/icd/code/M15-M19.html) | other arthritis |
|  | [M40-M54](https://www.icd-code.de/icd/code/M40-M54.html) | dorsopathies |
|  | [M60-M79](https://www.icd-code.de/icd/code/M60-M79.html) | soft tissue disorders |
|  | [M80](https://www.icd-code.de/icd/code/M80.-.html) | osteoporosis with pathological fracture |
|  | [M81](https://www.icd-code.de/icd/code/M81.-.html) | osteoporosis without pathological fracture |
|  | [M00-M99](https://www.icd-code.de/icd/code/M00-M99.html) | other diseases of the musculoskeletal system and connective tissue |
| Kidney | | |
|  | [N18](https://www.icd-code.de/icd/code/N18.-.html) | chronic kidney disease |
|  | [N20-N23](https://www.icd-code.de/icd/code/N20-N23.html) | urolithiasis |
|  | [N30](https://www.icd-code.de/icd/code/N30.-.html) | urinary tract infection |
|  | N39 | urinary incontinence |
|  | [N40](https://www.icd-code.de/icd/code/N40.html) | hyperplasia of prostate |
|  | [N80-N98](https://www.icd-code.de/icd/code/N80-N98.html) | noninflammatory disorders of female genital tract |
|  | [N00-N99](https://www.icd-code.de/icd/code/N00-N99.html) | other diseases of the genitourinary system |
| Pregnancy childbirth and puerperium | | |
|  | [O00-O99](https://www.icd-code.de/icd/code/O00-O99.html) | pregnancy, childbirth and the puerperium |
| perinatal period | | |
|  | [P00-P96](https://www.icd-code.de/icd/code/P00-P96.html) | certain conditions originating in the perinatal period |
|  | [Q00-Q99](https://www.icd-code.de/icd/code/Q00-Q99.html) | congenital malformations, deformations and chromosomal abnormalities |
| external causes | | |
|  | S00-T98 Z | injury, poisoning and certain other consequences of external causes |
|  | T88.7, X49, Y57 | drug reaction, adverse drug reactions, drug overdosing |
|  | T78.4 | unspecified allergies |
|  | [S00-T98](https://www.icd-code.de/icd/code/S00-T98.html) | other injury, poisoning and certain other consequences of external causes |
| special purposes | | |
|  | U07 | COVID-19 |
|  | [U00-U99](https://www.icd-code.de/icd/code/U00-U99.html) | other codes for special purposes |
| external causes of morbidity and mortality | | |
|  | V01-Y84 | external causes of morbidity and mortality |
