# Supplementary material for: Developing and testing a framework for coding general practitioners’ free-text diagnoses in electronic medical records - a reliability study for generating training data in natural language processing
Source: BMC Prim Care. 2024 Jul 16;25:257. doi: 10.1186/s12875-024-02514-1 (PMC11251376; doi:10.1186/s12875-024-02514-1)
Supplement: Supplementary file 2 — Supplementary Material 2 [file 12875_2024_2514_MOESM2_ESM.docx]

Additional file 2: Complete Frequency Analysis

| ICD-Origin | Code | Rater 1 | Rater 2 | Avg. of LoFT% | Kappa |
| --- | --- | --- | --- | --- | --- |
| no diagnosis | no diagnosis | 15300 | 15091 | 56.3% | 0.856 |
| M40-M54 | dorsopathies | 1056 | 1066 | 3.9% | 0.932 |
| I00-I99 | other diseases of the circulatory system | 824 | 865 | 3.1% | 0.848 |
| M00-M99 | other diseases of the musculoskeletal system and connective tissue | 769 | 758 | 2.8% | 0.743 |
| I10 | primary hypertension | 713 | 704 | 2.6% | 0.972 |
| S00-T98 | injury, poisoning and certain other consequences of external causes | 654 | 690 | 2.5% | 0.853 |
| D00-D48 | other neoplasms | 581 | 588 | 2.2% | 0.852 |
| E78 | disorders of lipoprotein metabolism and other lipidaemias | 545 | 539 | 2.0% | 0.985 |
| E00-E90 | other endocrine, nutritional and metabolic diseases | 489 | 501 | 1.8% | 0.876 |
| M60-M79 | soft tissue disorders | 415 | 463 | 1.6% | 0.734 |
| K00-K93 | other diseases of the digestive system | 414 | 449 | 1.6% | 0.786 |
| L00-L99 | other diseases of the skin and subcutaneous tissue | 401 | 458 | 1.6% | 0.833 |
| H00-H59 | diseases of the eye and adnexa | 344 | 350 | 1.3% | 0.9 |
| C00-C99 | malignant neoplasms | 333 | 359 | 1.3% | 0.839 |
| F17 | mental and behavioural disorders due to use of tobacco | 312 | 315 | 1.2% | 0.969 |
| I20-I25 | ischaemic heart diseases | 297 | 305 | 1.1% | 0.925 |
| K57 | diverticular disease of intestine | 284 | 281 | 1.0% | 0.973 |
| N00-N99 | other diseases of the genitourinary system | 252 | 302 | 1.0% | 0.78 |
| K21 | gastro-oesophageal reflux disease | 262 | 260 | 1.0% | 0.957 |
| E65-E68 | obesity and other hyperalimentation | 260 | 260 | 1.0% | 0.961 |
| G00-G99 | other diseases of the nervous system | 234 | 263 | 0.9% | 0.778 |
| F32-F33 | depressive episode and recurrent depressive disorder | 238 | 249 | 0.9% | 0.96 |
| E00-E07 | disorders of thyroid gland | 223 | 238 | 0.9% | 0.883 |
| J00-J99 | other diseases of the respiratory system | 212 | 247 | 0.9% | 0.751 |
| A00-B99 | intestinal infectious diseases | 236 | 218 | 0.8% | 0.785 |
| K40-K46 | intestinal hernia | 221 | 221 | 0.8% | 0.95 |
| H60-H95 | other diseases of the ear and mastoid process | 226 | 207 | 0.8% | 0.862 |
| E11 | type 2 diabetes mellitus | 217 | 211 | 0.8% | 0.906 |
| I83 | varicose veins of lower extremities | 195 | 217 | 0.8% | 0.882 |
| D50-D90 | other diseases of the blood and blood-forming organs and certain disorders involving the immune mechanism | 183 | 207 | 0.7% | 0.782 |
| T88.7, X49, Y57 | drug reaction, adverse drug reactions, drug overdosing | 194 | 192 | 0.7% | 0.765 |
| M17 | arthritis of the knee | 183 | 176 | 0.7% | 0.952 |
| F40-F48 | neurotic, stress-related and somatoform disorders | 162 | 197 | 0.7% | 0.839 |
| J45 | asthma | 174 | 177 | 0.7% | 0.945 |
| I11-I14 | hypertension with end organ damage | 163 | 159 | 0.6% | 0.962 |
| T78.4 | unspecified allergies | 139 | 169 | 0.6% | 0.765 |
| G47 | sleep disorders | 150 | 147 | 0.6% | 0.949 |
| F00-F99 | other mental and behavioural disorders | 146 | 148 | 0.5% | 0.781 |
| K29 | gastritis and duodenitis | 143 | 150 | 0.5% | 0.914 |
| I48 | atrial fibrillation and flutter | 147 | 140 | 0.5% | 0.961 |
| I60-I69 | cerebrovascular diseases | 135 | 130 | 0.5% | 0.874 |
| N80-N98 | noninflammatory disorders of female genital tract | 133 | 128 | 0.5% | 0.896 |
| K64 | haemorrhoids and perianal venous thrombosis | 122 | 119 | 0.4% | 0.921 |
| E55 | vitamin D deficiency | 112 | 113 | 0.4% | 0.96 |
| N40 | hyperplasia of prostate | 106 | 115 | 0.4% | 0.95 |
| N18 | chronic kidney disease | 110 | 108 | 0.4% | 0.954 |
| G43 | migraine | 98 | 105 | 0.4% | 0.955 |
| M18-M19 | other arthritis | 107 | 96 | 0.4% | 0.777 |
| I87 | other disorders of veins | 99 | 100 | 0.4% | 0.924 |
| Q00-Q99 | congenital malformations, deformations and chromosomal abnormalities | 107 | 85 | 0.4% | 0.728 |
| M81 | osteoporosis without pathological fracture | 84 | 92 | 0.3% | 0.909 |
| I70 | atherosclerosis | 80 | 96 | 0.3% | 0.772 |
| J44 | other chronic obstructive pulmonary disease | 88 | 84 | 0.3% | 0.918 |
| J30 | vasomotor and allergic rhinitis | 105 | 65 | 0.3% | 0.729 |
| N20-N23 | urolithiasis | 83 | 83 | 0.3% | 0.903 |
| M16 | arthritis of the hip | 83 | 82 | 0.3% | 0.921 |
| K80-K87 | disorders of gallbladder, biliary tract and pancreas | 83 | 81 | 0.3% | 0.865 |
| K70-K77 | diseases of liver | 78 | 74 | 0.3% | 0.855 |
| N30 | urinary tract infection | 73 | 75 | 0.3% | 0.89 |
| J00-J06 | acute upper respiratory infections | 93 | 53 | 0.3% | 0.502 |
| M80 | osteoporosis with pathological fracture | 69 | 74 | 0.3% | 0.769 |
| M15 | polyarthritis | 70 | 68 | 0.3% | 0.811 |
| O00-O99 | pregnancy, childbirth and the puerperium | 65 | 67 | 0.2% | 0.803 |
| G40 | epilepsy | 63 | 64 | 0.2% | 0.882 |
| I34 | nonrheumatic mitral valve disorders | 56 | 63 | 0.2% | 0.839 |
| J09-J18 | influenza and pneumonia | 58 | 55 | 0.2% | 0.885 |
| F10 | mental and behavioural disorders due to alcohol | 55 | 56 | 0.2% | 0.828 |
| E14 | unspecified diabetes mellitus | 51 | 56 | 0.2% | 0.822 |
| I50 | heart failure | 42 | 64 | 0.2% | 0.754 |
| D50 | iron deficiency anaemia | 46 | 51 | 0.2% | 0.804 |
| F10-F19 | mental and behavioural disorders due to psychoactive substance use | 46 | 50 | 0.2% | 0.893 |
| F03 | unspecified dementia | 47 | 47 | 0.2% | 0.911 |
| M10 | gout | 47 | 42 | 0.2% | 0.943 |
| H81 | disorders of vestibular function | 44 | 44 | 0.2% | 0.839 |
| G60-G64 | polyneuropathies and other disorders of the peripheral nervous system | 48 | 40 | 0.2% | 0.795 |
| I15 | secondary hypertension | 45 | 40 | 0.2% | 0.94 |
| N39 | urinary incontinence | 42 | 42 | 0.2% | 0.809 |
| G20-G26 | parkinson's disease | 34 | 40 | 0.1% | 0.865 |
| K50-K52 | noninfective enteritis and colitis | 37 | 37 | 0.1% | 0.783 |
| F20-F29 | schizophrenia, schizotypal and delusional disorders | 33 | 32 | 0.1% | 0.769 |
| G45 | transient cerebral ischaemic attacks and related syndromes | 30 | 32 | 0.1% | 0.806 |
| G44 | other headache syndromes | 27 | 35 | 0.1% | 0.677 |
| L40 | psoriasis | 31 | 30 | 0.1% | 0.918 |
| K25-K28 | peptic ulcer | 27 | 30 | 0.1% | 0.877 |
| B02 | herpes zoster | 30 | 26 | 0.1% | 0.928 |
| K58 | irritable bowel syndrome | 27 | 29 | 0.1% | 0.928 |
| E05 | hyperthyroidism | 31 | 24 | 0.1% | 0.873 |
| B15-B19 | viral hepatitis | 26 | 24 | 0.1% | 0.84 |
| U00-U99 | codes for special purposes | 21 | 28 | 0.1% | 0.448 |
| H66 | suppurative and unspecified otitis media | 25 | 19 | 0.1% | 0.864 |
| F30-F39 | affective disorders | 20 | 22 | 0.1% | 0.857 |
| A00-A09 | other infectious and parasitic diseases | 21 | 21 | 0.1% | 0.476 |
| F50 | eating disorders | 16 | 19 | 0.1% | 0.848 |
| G35 | multiple sclerosis | 12 | 14 | 0.0% | 0.846 |
| V01-Y84 | external causes of morbidity and mortality | 9 | 11 | 0.0% | 0.3 |
| E10 | type 1 diabetes mellitus | 7 | 12 | 0.0% | 0.631 |
| B00 | herpes simplex infections | 10 | 6 | 0.0% | 0.75 |
| K30 | functional dyspepsia | 8 | 7 | 0.0% | 0.533 |
| P00-P96 | certain conditions originating in the perinatal period | 7 | 7 | 0.0% | 1 |
| G20 | extrapyramidal and movement disorders | 5 | 6 | 0.0% | 0.909 |
| J02 | acute pharyngitis | 7 | 3 | 0.0% | 0.6 |
| J20-J22 | other acute lower respiratory infections | 4 | 2 | 0.0% | 0 |
| H65 | nonsuppurative otitis media | 1 | 2 | 0.0% | 0.667 |
| B20-B24 | human immunodeficiency virus disease | 1 | 1 | 0.0% | 1 |
| U07 | COVID-19 | 1 | 1 | 0.0% | 0 |
